# Shifting the Redox-Flow Battery Trade-Off with Amine-Crosslinked PVBC Thin-Film Composite Membranes

**DOI:** 10.3390/membranes16090291

**Published:** 2026-08-31

**Authors:** Chiari Van Cauter, Maarten Cools, Yun Li, Ivo F. J. Vankelecom

**Affiliations:** Membrane Technology Group (MTG), Division cMACS, Faculty of Bioscience Engineering, KU Leuven, Celestijnenlaan 200F, Box 2454, 3001 Leuven, Belgium

**Keywords:** vanadium flow battery, thin film composite, membrane, interfacial crosslinking, PVDF

## Abstract

Redox flow batteries (RFBs) are an interesting option for long-term energy storage. A well-performing membrane sits at the heart of the electrochemical battery cell and should effectively mitigate crossover of active species while minimizing resistance. However, current commercial membranes are rather expensive and demonstrate sub-optimal performance, leading to an extensive search for alternatives. Research on membranes for RFBs has long been dominated by dense ion-exchange membranes and porous membranes, both potentially with fillers. In recent years, increased interest in alternative morphologies such as thin-film composites (TFCs) has ignited new research directions. TFCs consist of a thin dense layer on top of a porous support, aiming to merge the advantages of both. Traditionally, TFCs are made using polyamide top layers. In this paper, a novel chemistry is developed with increased chemical stability for RFBs. Poly(vinylbenzyl chloride) is crosslinked interfacially with a diamine, demonstrating for the first time the potential of support-mediated interfacial crosslinking with two immiscible solvents. Optimization of the support, amine crosslinker, reaction time and synthesis procedure allowed a shift of the trade-off between vanadium crossover and proton transport, highlighting the opportunities for this promising TFC chemistry.

## 1. Introduction

The increasing share of renewable energy requires large-scale energy storage to safeguard grid stability. One option is lithium-ion batteries, which are better at providing short-term storage instead of long-term due to cost inefficiencies at a larger scale [1,2]. Redox flow batteries (RFBs) are an optimal candidate for stationary energy storage due to their decoupled power and capacity and long lifetime. This makes them more cost-efficient at a larger scale. However, one of the remaining drawbacks is their relatively high price compared to conventional energy storage devices as well as their sub-optimal performance. The membrane is an essential cell component that significantly dictates the performance. Dense ion exchange membranes are the default, with perfluorosulfonic chemistries such as in Nafion being the benchmark [3,4,5]. However, such dense ion exchange membranes typically suffer from a trade-off between conductivity and selectivity due to their morphology. Here, a thin-film composite (TFC) membrane is developed as an alternative membrane to improve the selectivity without compromising ionic conductivity.

A TFC consists of a porous support and a very thin, dense and positively charged layer on top to seal the pores of the underlying support. TFCs have long been known in the membrane field and have immensely improved liquid filtration [6,7], but only recently have they made their way into the RFB field [8,9]. Here, an improved conductivity can be obtained by the ultra-thin and highly charged top layer combined with the high porosity of the support, while the crossover of redox-active species can be suppressed upon developing a sufficiently dense top layer.

TFC morphologies have been exploited to minimize the amount of perfluorinated polymer present in the membrane [10]. This allows for application-tailored optimization by separate tuning of the support and top layer, with many polymer chemistries available for both. Most commonly known is the TFC with a polyamide top layer, already demonstrated in flow batteries [8,11]. Although Dai et al. showed a stable operation for 1000 cycles [8], polyamide is theorized to be unstable in acid conditions on the longer term but can be further stabilized through the addition of other polymers. This is exemplified by the addition of a polydopamine layer by Teng et al. [9]. Alternatively, different thin-film chemistries are explored, such as polypyrrole [12], polybenzimidazole [13,14], layered double hydroxide [15] and polymers of intrinsic microporosity [16,17].

A composite membrane is now introduced based on a poly(vinylidene fluoride) (PVDF) support and an amine-crosslinked poly(vinylbenzyl chloride) (PVBC) top layer with quaternary ammonium groups. Although other variations of so-called interfacial crosslinking exist [18,19,20,21,22,23], this is the first time that a polymer/oligomer is crosslinked interfacially on a porous support by the use of two immiscible solvents. This interfacial crosslinking allows for obtaining much thinner polymer films than conventional polymer crosslinking. The latter is done before polymer solution casting [24], during phase inversion [25] or after casting as a post-treatment [26,27]. In all cases, membranes for RFBs are generally cast as free-standing, which requires a higher thickness in order to be mechanically stable. Performing the reaction interfacially allows the formation of a thin layer of crosslinked polymer, which is anchored in the porous support. The commercial PVDF support Nadir UV150 is compared to a lab-made PVDF support, which was optimized by tuning synthesis parameters such as polymer concentration and post-treatment. The PVBC top layer is modified by varying crosslinker and reaction parameters, such as reactant concentration, reaction time and synthesis procedure.

The bifunctional amines 1,4-diazabicyclo[2.2.2]octane (DABCO) and N,N,N’,N’-tetramethyl-1.6-hexanediamine (TMHDA) are used to incorporate positive charge into the top layer through crosslinking. Both are tertiary amines, creating a quaternary ammonium upon reacting with the chloromethyl moiety of PVBC according to a Menshutkin reaction [28,29], following an S_N_2 mechanism [30]. DABCO and TMHDA have both been used for charge incorporation and as a crosslinker to create high-performance anion exchange membranes (AEMs) [31,32,33,34]. Though they are mostly used in an alkaline environment for high OH^-^ conductivity [29,35,36,37,38,39,40,41,42], few studies also prove their potential in acid-based RFBs [27,33,43,44,45].

Besides their stability, the general effect of quaternary ammonium groups on the performance of the membrane has also been studied. Rezayani et al. found that putting the charge on the end of the pending chain rather than at the beginning did not alter the shape of the microphase but changed the mobility of anions through the membrane [46]. Additionally, they also found that increased hydrophobic side chain length dangling on a quaternary ammonium group resulted in steric hindrance and lowered the mobility of anions through the membrane [46]. Li et al. found that amines with longer alkyl chains, such as TMHDA compared to trimethylamine, resulted in higher chemical stability, attributed to increased steric hindrance [39]. Komkova et al. similarly found a higher stability with longer alkyl chains [40].

## 2. Materials and Methods

### 2.1. Materials

Poly(vinylidene fluoride) (PVDF) (MW ~ 534 000 Da), poly(vinylbenzyl chloride) (PVBC) (60/40 mixture of 3- and 4-isomers), magnesium sulfate (MgSO_4_), 1,6-dibromohexane and deuterium oxide (99.9%) were purchased from Sigma Aldrich (St Quentin Fallavier, France). Toluene (>99%), vanadyl sulfate hydrate and ethanol (>99%) were purchased from Fischer Scientific (Kandel, Germany), 1,4-diazabicyclo[2,2,2]octane (DABCO) (>98%) and tetramethyl hexane diamine (TMHDA) from TCI Europe (Zwijndrecht, Belgium), dimethyl sulfoxide (DMSO) (99.9+%) from Alfa Aesar (ThermoFischer, Kandel, Germany), potassium chloride (KCl) (99.995%) and potassium hydroxide (KOH) (99.98%) from Thermo Scientific (Kandel, Germany), ethanol (97%) from Chemlab NV (now called ‘AnalytiChem Belgium NV’, Zedelgem, Belgium), sulfuric acid (H_2_SO_4_) (96%) from Acros Organics (Geel, Belgium), hydrogen chloride (HCl) (37%) from VWR (Radnor, PA, USA), Hollytex 3329 from Ahlstrom (Espoo, Finland) and Nadir UV150 from Microdyn Nadir GmbH (Mann+Hummel, Ludwigsburg, Germany). Commercial ion-exchange membranes Nafion™ 115 (dry thickness 127 µm) and FAP 450 (dry thickness 50 µm) were purchased from Sigma-Aldrich and Redox-Flow (Risskov, Denmark), respectively. Prior to use, both membranes were immersed in 0.5 M H_2_SO_4_ for 48 h.

### 2.2. Methods

#### 2.2.1. Membrane Synthesis

##### Support

Supports were either commercial Nadir UV150 or lab-made by dissolving 12–20 wt% PVDF in DMSO and stirring at 60 °C for 1 day. The solution was left to rest for 1 night at 60 °C and was subsequently degassed in vacuum for 3 h to minimize pinhole formation. For polymer concentrations of 12–15 wt%, this was done at room temperature. For polymer concentrations of 16–20 wt%, this was done at 50 °C to maintain easily castable viscosities. The polymer solution was cast immediately with an automatic casting machine (MEMCAST^TM^, Porometer, Nazareth-De Pinte, Belgium) with a doctor blade of 250 µm thickness on a glass plate at speed 2 cm s^−1^ and was immersed in a room temperature deionized (DI) water bath for 20 min. Afterwards, the support was stored in DI water in a refrigerator at 6 °C until further use.

Optionally, the support was crosslinked as described by Van Goethem et al. [26]. This was done by immersing the support in MeOH for 10 min, after which it was immersed in an HDA solution containing MgO at 60 °C, for 15 min–1 h. After crosslinking, the membranes were rinsed with MeOH and stored in DI water until further use.

##### Synthesis of DABCO-Oligomer

DABCO-oligomer was synthesized in a three-neck round bottom flask reacting 1,6-dibromohexane with DABCO, as described by Turrina et al. [47]. A total of 9.6 g DABCO was dissolved in 30 mL ethanol, and a solution of 2 v% 1,6-dibromohexane in ethanol was added dropwise under continuous stirring. The reaction mixture was heated to 50 °C (oil bath) and refluxed for 24 h. Afterwards, the mixture was cooled down to 20 ± 2 °C, and the ethanol was removed with a rotary evaporator until only a white powder remained. The resulting white powder was washed with diethyl ether at room temperature and dried overnight at 50 °C. From the recipe, the majority of DABCO-oligomers were expected to have a pentamer length (being 5x DABCO linked by 4 hexane molecules) (Turrina et al. had a 89% yield), thus the MW of this oligomer length was used for concentration calculations. However, NMR results show that here, the actual length was closer to a nonamer (9 DABCO linked by 8 hexane molecules) (Figure S1).

##### Viability of Top-Layer Reaction

To evaluate the possibility of two reagents forming a top layer at the interface of two immiscible solvents, vial tests were performed. Here, an amine was dissolved in DI water and brought in contact with PVBC dissolved in toluene in a glass vial. Water has the highest density and was inserted into the vial first; toluene has a lower density and was calmly added to the vial without disturbing the forming interface. No support was present, so the contact of the two reagents was not limited by mass transport through the support. For DABCO-oligomer as the chosen amine, the vials were tested both at room temperature and at 50 °C. Different concentrations of PVBC and DABCO-oligomer were tested both at room temperature and at 50 °C. Experiments with amine variations (DABCO-oligomer, DABCO and TMHDA) were all performed at 50 °C.

##### Composite Membrane

**Figure 1:**
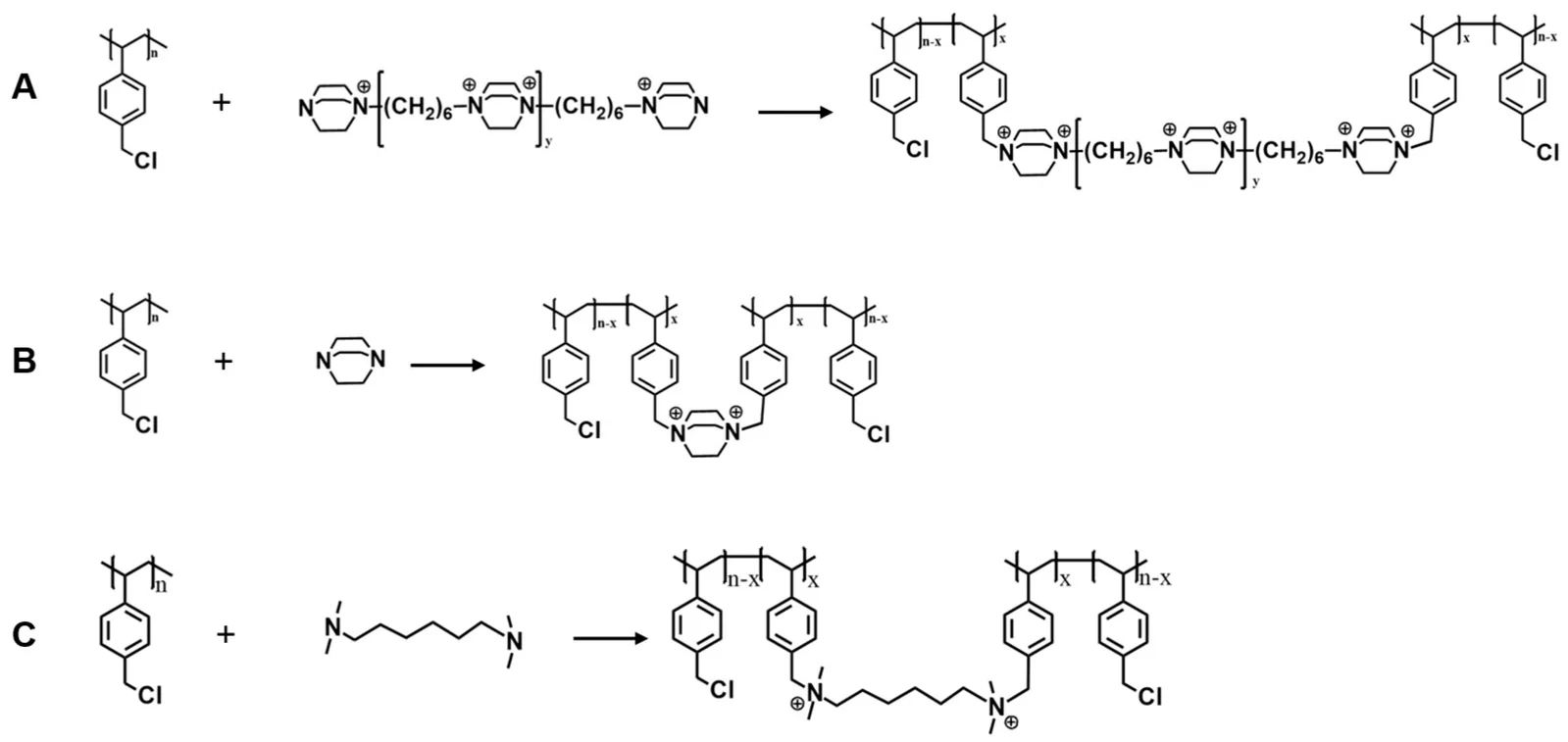
Top-layer film-formation reaction of PVBC with (**A**) DABCO-oligomer, (**B**) DABCO and (**C**) TMHDA.

The support was immersed in an amine/water solution for 1 to 1.5 h. This amine/water solution generally consisted of either 10 wt% DABCO-oligomer, 0.83 wt% DABCO or 1.41 wt% TMHDA. After support immersion in the aqueous solution, the remaining droplets were removed from the surface before clasping the support in a lab-made interfacial cell. A PVBC/toluene solution (generally 2.5 wt%) was added on top, and the complete set-up was put in an oven (Binder FD 53, Binder GmbH, Tuttlingen, Germany) at 50 °C with a lid to avoid excessive evaporation. The envisioned top layer reactions are depicted in Figure 1. After varying times (30 min–4 h), the set-up was taken from the oven, the PVBC/toluene solution was discarded and the membrane was washed with toluene and ethanol to remove unreacted PVBC. Optionally, a second crosslinking step was performed by applying an amine/water solution to the membrane surface after removal of excess PVBC. Subsequently, the reaction was allowed to proceed in the oven. After the prescribed reaction time, the membrane was rinsed thoroughly with DI water to remove unreacted residual amine. The membrane was subsequently stored in DI water until further use.

#### 2.2.2. Area Ohmic Resistance

**Figure 2:**
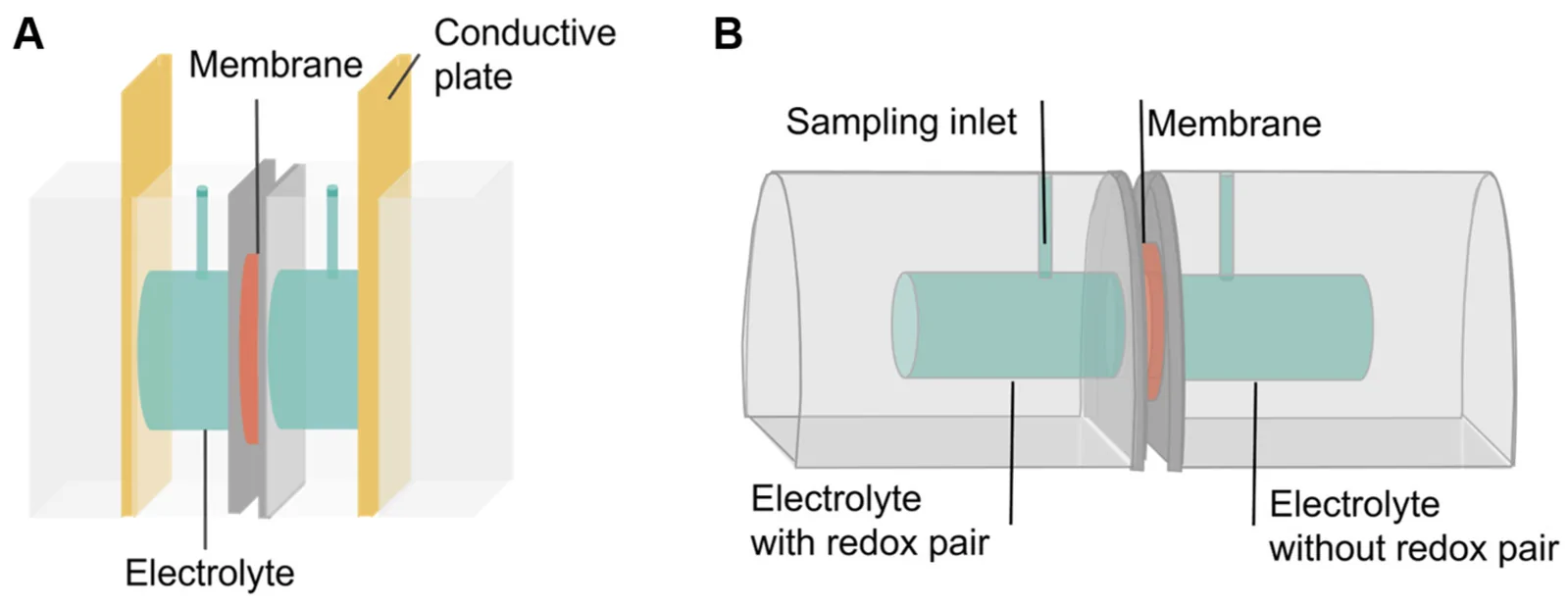
Scheme of (**A**) lab-made resistance cell and (**B**) lab-made diffusion cell.

The membrane area ohmic resistance (AOR) was measured with potentiostatic electrochemical impedance spectroscopy (PEIS)(BioLogic SAS SP-200, Seyssinet-Pariset, France) after 2 days of storage in 0.5 M H_2_SO_4_. The membrane was clasped in a lab-made resistance cell with 0.5 M H_2_SO_4_ solution and copper electrodes on each side of the membrane (Figure 2A). Resistances were read from the low-frequency intercept of the Nyquist plot and were compared to the resistance of a membrane-free cell, according to Equation (1):

(1) $$AOR=\left(r_{m}-r_{s}\right)\times A$$

where *r_m_* is the resistance of the cell with the membrane and electrolyte solution, *r_s_* is the resistance of the cell containing only the electrolyte solution, and *A* is the active surface of the electrode and membrane.

#### 2.2.3. Diffusion Coefficient

The crossover is measured in a lab-made diffusion cell (Figure 2B). The membrane is clasped in the middle, and a solution with a redox-pair is added on one side, while an osmotically balanced solution without a redox pair is added on the other side. Both sides are stirred to minimize concentration polarization. Samples are taken from the non-redox pair side, through the vertical inlet, at set time intervals, and the concentration of the redox pair is measured through UV–Vis. From this, the diffusion coefficient of the redox pair through the membrane can be measured according to the following formula (Equation (2)):

(2) $$J=-D\frac{\partial c}{\partial x}↔D=\frac{-Vx\ln\frac{c_{0}-c_{t}}{c_{0}}}{A\Delta t}$$

where *V* is the volume of the diffusion cell, *x* is the thickness of the membrane, *c*_0_ is the initial concentration of the redox pair, *c_t_* is the concentration at time of measurement, *A* is the active surface area of the membrane, and Δ*t* is the time passed between the start of the experiment and the measurement. In essence, a minimum of 5 samples were taken and plotted in $\ln\frac{c_{0}-c_{t}}{c_{0}}$ vs. time. The slope was then multiplied with $\frac{-Vx}{A}$ to obtain the diffusion coefficient. When reporting the crossover of the composite membranes, permeances are reported instead of thickness-normalized permeabilities, to account for the multi-layered structure and the different thicknesses of composite layers.

#### 2.2.4. Selectivity

The membrane selectivity (S, mS.s cm^−3^) was calculated using the formula below:

(3) $$S=\frac{\sigma }{P}$$

where *σ* is the conductivity (mS cm^−1^) and *P* is the vanadium permeability (cm^2^ s^−1^).

#### 2.2.5. Physicochemical Characterization

##### Scanning Electron Microscopy (SEM)

Membrane morphology was characterized with SEM (JEOL JSM-6010LV, JEOL Europe, Zaventem, Belgium). The membranes were dried overnight, cryofractured in liquid nitrogen and coated with an Au/Pd conductive layer (JEOL JFC-1300 Auto Fine Coater, JEOL Europe, Zaventem, Belgium) before measurement.

##### Proton Nuclear Magnetic Resonance (^1^H-NMR)

Liquid-state ^1^H-NMR was performed to determine the average length of the DABCO-oligomer crosslinker. A solution with a concentration of 20 mg/mL in D_2_O was made, of which 0.5 mL was put in an NMR vial and tested with a Bruker Avance II+ 600 spectrometer. Data analysis was performed with Bruker TopSpin software (version 4.5).

##### Zeta Potential

Zeta potential was measured on Anton Paar SurPASS 3 (Anton Paar GmbH, Gentbrugge, Belgium). The membrane samples were stored in milli-Q water before measurement. After blotting the support side of the membrane, the samples were cut in 2 cm × 1 cm rectangles and attached to the sample holder with carbon tape. Each measurement was performed in 150 mL of 1 mM KCl solution (in milli-Q water). This solution was first acidified to pH 2 by using a 0.1 M HCl solution. The electrolyte solution was purged with argon gas to remove dissolved CO_2_, which would cause a buffering effect and consequent pH drift. Prior to the pH scan, 10 rinse steps at 600–200 mbar with a gap height between 90 and 110 µm were performed to allow the sample to stabilize in the acidified electrolyte solution. The pH scan was initiated once the sample was stabilized. Each step involved 2 rinse cycles with 4 subsequent zeta potential measurements (600–200 mbar, values > 500 mbar are omitted to reduce measurement errors). The pH was increased with steps of 1.5 by using a 0.1 M KOH solution.

##### Swelling Ratio

The swelling ratio of supports was measured by cutting support strips at a predetermined length (4 cm × 1 cm) and storing them in the swelling solvent, i.e., toluene, during 3 days at room temperature. Afterwards, the swollen length was measured, and the swelling ratio was calculated according to Equation (4):

(4) $$Swelling\;ratio\;\left(\%\right)=\frac{L_{1}-L_{0}}{L_{0}}$$

where *L*_1_ is the length after toluene storage and *L*_0_ is the length before toluene storage. A high swelling ratio may indicate insufficient stability in the chosen solvent.

##### Infrared Spectroscopy

Attenuated Total Reflectance—Fourier Transformed Infared (ATR-FTIR) spectroscopy was used to check the chemical composition of the membrane, mainly in relation to top-layer formation. A Bruker Alpha II Spectrometer (Bruker Alpha, Karlsruhe, Germany) with diamond crystal was used. Software OPUS 7.5 was used, and 32 scans were taken at a resolution of 2 cm^−1^ in the range of 400–4000 cm^−1^.

## 3. Results and Discussion

Vial tests of PVBC and DABCO-oligomer indicated limited to no reactivity at room temperature, even after several days. A temperature of 50 °C was necessary to speed up the reaction, which aligns with the endothermic behavior shown in simulations by other researchers [30]. To eliminate reaction temperature as a parameter, all interfacial reactions were performed at 50 °C.

### 3.1. Confirmation of Crosslinked Top Layer

**Figure 3:**
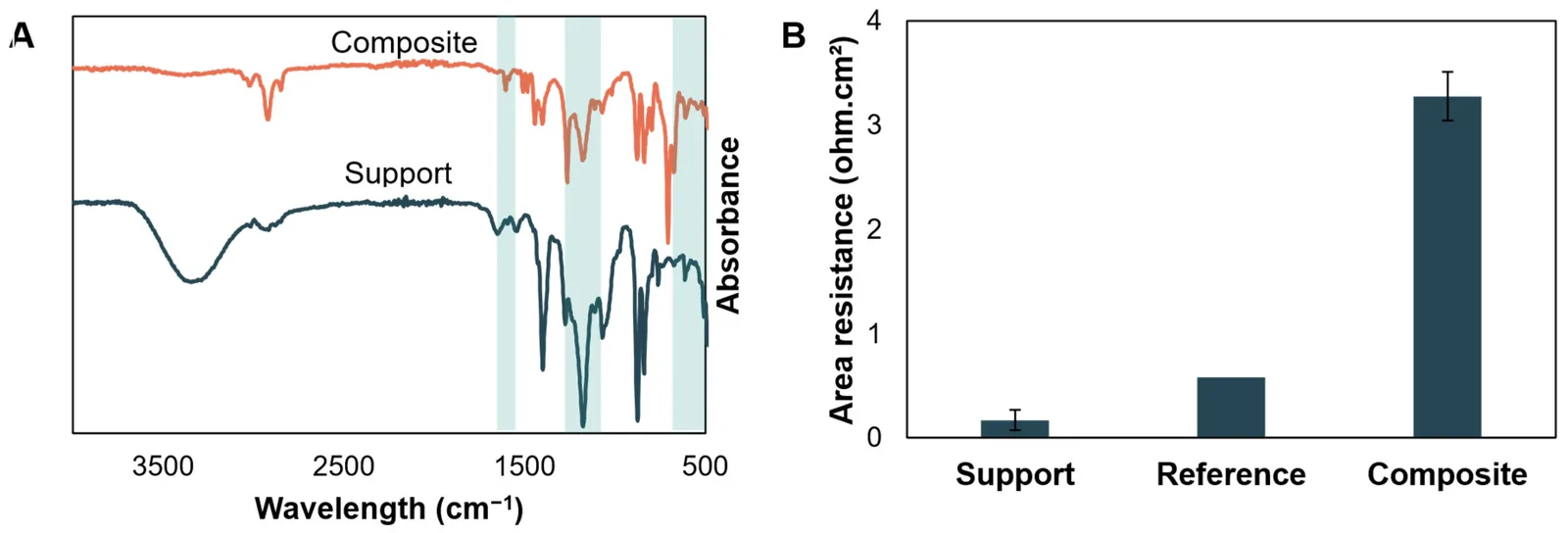
(**A**) ATR-FTIR spectra of pristine Nadir UV150 support and composite membrane made from PVBC and DABCO-oligomer. The discussed changes are highlighted for ease of reading. (**B**) Area resistance of composite membrane made from PVBC and TMHDA compared to the support and a reference membrane made according to the same procedure but lacking amine in the aqueous phase during synthesis.

The presence of the top layer is confirmed through ATR-FTIR spectroscopy. Figure 3A shows the spectra of both the Nadir support and the TFC formed on Nadir. The spectra look vastly different, confirming that a reaction took place. The crosslinking of the top layer is difficult to confirm, as quaternary ammonia groups lack N-H bonds for detection [48]. C-N bonds can be read from the infrared spectra, but do not indicate whether the amine crosslinker reacted only once or formed a crosslink. Instead, only the presence of a general top layer, based on PVBC and amine, can be confirmed. Specifically, two peaks at 625–755 cm^−1^ indicate the presence of C-H out-of-plane bending, as present in aromatic structures. PVBC is the only molecule present containing aromatic structures, thus confirming its presence in the top layer. Additionally, 1267 cm^−1^ and 1176 cm^−1^ peaks can be attributed to C-N stretching [48,49], indicating the presence of the amine-based crosslinker. The extent of crosslinking could not be quantified using the CH_2_-Cl wagging band at 1266 cm^−1^, as this band overlaps with the C-N stretching vibration [50]. A small peak at 1603 cm^−1^ could indicate the presence of protonated amine, having only reacted on one side.

To confirm whether the top layer was merely a deposition of PVBC on support or truly a crosslinked polymer film, a reference membrane was made, and the area resistance was quantified. The reference procedure consisted of the same procedure as for top layer formation but without amine in the aqueous phase. Should the top layer consist of merely deposited PVBC, the area resistance of assumed PVBC/amine composite would be similar to the reference. In Figure 3B, the area resistance is depicted. The reference without amine shows little difference from the support, whilst a clear difference is seen when both PVBC and amine are present in their respective phases during the interfacial reaction, proving that the top layer is the result of a crosslink between PVBC and amine.

### 3.2. Effect of Support

#### 3.2.1. Commercial vs. Lab-Made: The Effect of Non-Woven Supports

**Figure 4:**
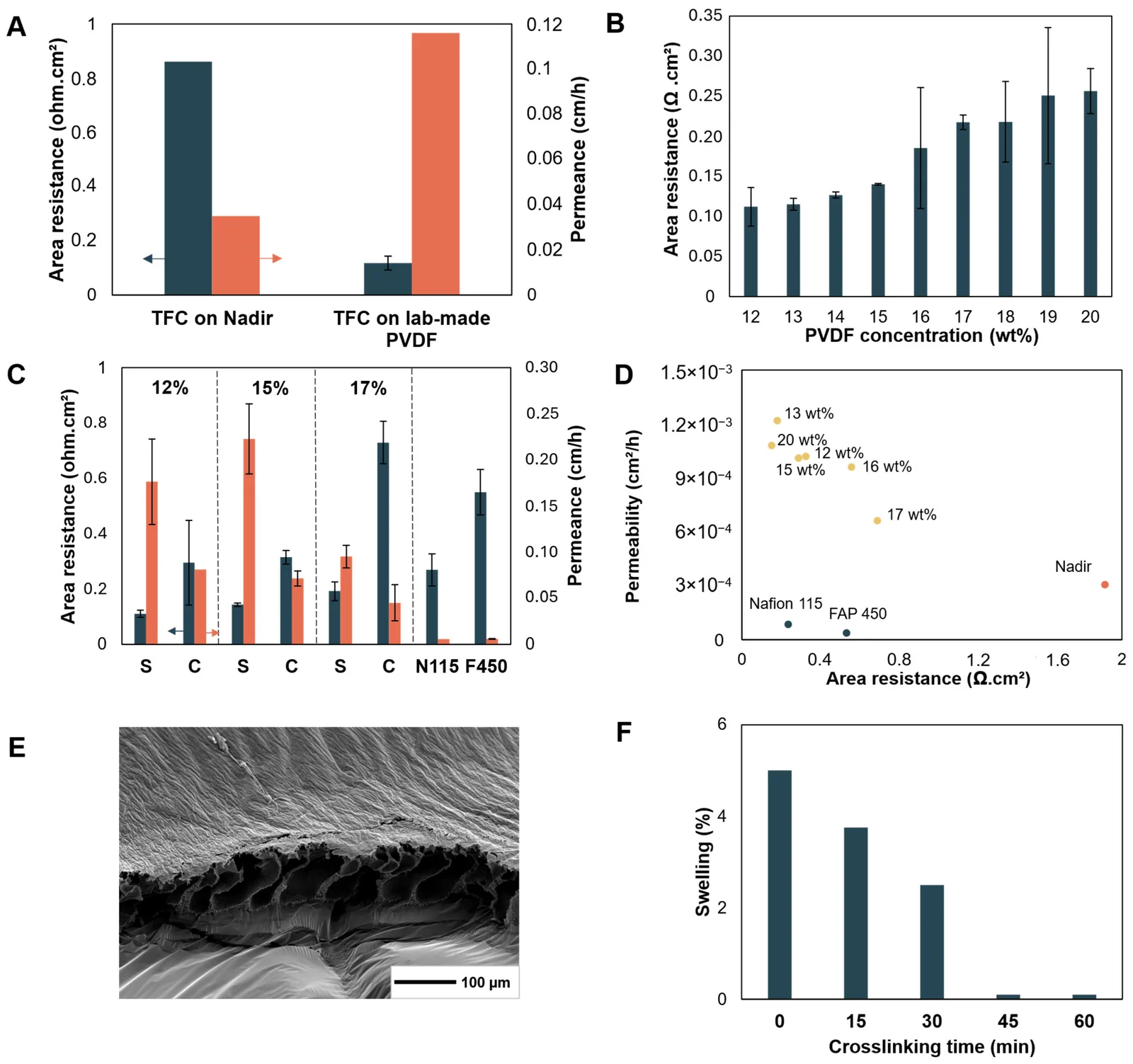
(**A**) Area resistance (blue, **left**) and vanadium permeance (red, right) of thin-film composites made on Nadir UV150 and lab-made PVDF, 12 wt%. (**B**) Area resistance of porous support membranes made from increasing PVDF concentrations (12–20 wt%) in the casting solution. (**C**) Area resistance (blue, **left**) and vanadium permeance (red, **right**) of thin-film composites (‘C’) made on supports prepared from varying PVDF concentration (12 wt%, 15 wt%, 17 wt%) compared to their respective supports (‘S’) and commercial membranes Nafion 115 and FAP 450. All measurements were performed in 0.5 M H_2_SO_4_. (**D**) Area resistance and permeability of thin-film composites on lab-made PVDF supports with varying PVDF concentration and on commercial support Nadir UV 150, compared to commercial membranes Nafion 115 and FAP 450. (**E**) SEM picture of lab-made PVDF support. (**F**) Swelling in toluene of lab-made PVDF membranes at different post-treatment crosslinking times.

The commercial support Nadir UV150 consists of a PVDF layer on a polyester non-woven support backing (Figure S2A) [51]. While the membrane allows for successful formation of a composite, the resulting area resistances are rather high and would not allow for competitive membrane development. Instead, a support with a more open and less tortuous path for ion conduction is needed. SEM images show a very open pore structure for Nadir UV150, on top of the fibrous non-woven polyester support (Figure S2B). The high area resistance of the commercial membrane is likely due to this polyester support [52]. Therefore, low resistance PVDF supports were developed. Even though diffusional crossover was higher compared to Nadir UV150 (Figure 4A), the lab-made support is still considered the more promising option since the selectivity of the TFC will be governed by the top layer.

#### 3.2.2. Polymer Concentration

The PVDF concentration in the casting solution was varied in the range of 12–20 wt%. At higher concentrations, the increased solution viscosity complicated casting. Therefore, dope solutions of 16 wt% and upwards were cast at an elevated temperature of 50 °C. In Figure 4B, the effect of PVDF concentration on the area resistance of the resulting support membrane is shown. The area resistance increases linearly, with all tested lab-made support membranes having an area resistance in the range of 0.1–0.25 ohm.cm^2^. Such low resistances are necessary to obtain a final membrane with high voltage efficiency. For comparison, Dai et al. employed a porous poly(ether sulfone)/sulfonated poly(ether ether ketone) (PES/SPEEK) membrane as support, reporting an area resistance of 0.11 Ω cm^2^ in 0.5 M H_2_SO_4_ [8]. The increase in resistance when increasing polymer concentration is often caused by a decreasing pore size and surface porosity. Indeed, the pore size of the support also has an effect on the top layer formation. Large pores in the skin layer can result in a relatively large area on which the top layer remains unsupported, which can result in a higher chance for defects in the top layer on low-concentration supports. However, the pore size distribution of the PVDF supports could not be measured through gas-liquid porometry due to the pronounced swelling of PVDF in the fluorinated wetting liquid. On the other hand, pore size can also influence the top layer formation during the interfacial synthesis. For example, in polyamide top layer systems, the pore is described as a hotspot for reaction, with the particular pore morphology affecting the roughness of the polyamide top layer [53].

The influence of different PVDF concentrations to prepare the supports is depicted in Figure 4C. Composite membranes on 15 wt% support show little improvement in vanadium permeance compared to composites on the 12 wt% supports. However, a 17 wt% support leads to a lower permeance. This is at the cost of an increased resistance, showcasing that composites from 15 wt% may have had too many uncovered support pores. The support of 17 wt% had the lowest vanadium permeability among all tested supports in the 12–20 wt% range (Figure 4D), even with its large pores in the substructure (Figure 4E). Increased polymer concentrations typically result in skin layer densification, allowing for an improved top layer formation. Too large pore sizes can limit the stability of the interfacial top layer, which can be visualized as bridging the pore walls. However, this effect is not endless. At increased polymer concentrations for preparing the supports, top layers may stagnate in crossover/rejection [54,55,56] past a certain threshold (17 wt% in this case) while showcasing lower flux/increased resistance. Even more, an increased crossover may again be visible [53]. Here, it is hypothesized that the transport of the amine crosslinker to the interface may be hindered at higher support polymer concentrations. Consequently, the effective concentration of the amine crosslinker at the interface is lower, which may result in a less crosslinked top layer, as showcased by the inferior performance of the TFC prepared using a 20 wt% support.

#### 3.2.3. Crosslinking of Supports

Another way in which the support can influence the composite is through its swelling behavior. Swelling of the support, especially when at a different rate than swelling of the top layer, may cause defects in the top layer. Whilst PVDF does not readily swell in aqueous solution, swelling is pronounced in toluene. Here, toluene is used during the synthesis of the top layer as a solvent for PVBC, and swelling of the PVDF support is noted almost immediately upon contact within the organic phase. Specifically, small ripples form within 5–30 s and stay constant once formed. To address suppression of the swelling of the support, crosslinking of PVDF was tested. Using a one pot procedure [26], PVDF was crosslinked with hexyl diamine (HDA) during different reaction times ranging from 15 min to 1 h, resulting in dark brown membranes (Figure S3). Decreased swelling at higher crosslinking times can be noted (Figure 4F), with a negligible swelling at 45 min crosslinking time. However, the mechanical stability of the supporting membrane also decreases with increasing crosslinking time. With substantial crosslinking times, i.e., starting at 30–45 min, the mechanical stability limited successful top layer formation, as handling became increasingly difficult. Synthesized membranes could not be fully tested due to tearing of the samples. Without a supporting layer, such as a non-woven support, the poor mechanical stability of the crosslinked PVDF does not allow use of this material as support.

Pristine PVDF is thus kept as the default support. While the swelling of PVDF is not ideal, timescales of swelling (within minutes) and top layer formation (hours) are considered sufficiently different to not affect top layer formation. Reduced swelling through another support polymer or another solvent could also be a viable mitigation option, but this is not discussed in this paper. Future research will focus on finding a different solvent for interfacial synthesis.

### 3.3. Effect of Top Layer

#### 3.3.1. Addition of Co-Solvent

**Figure 5:**
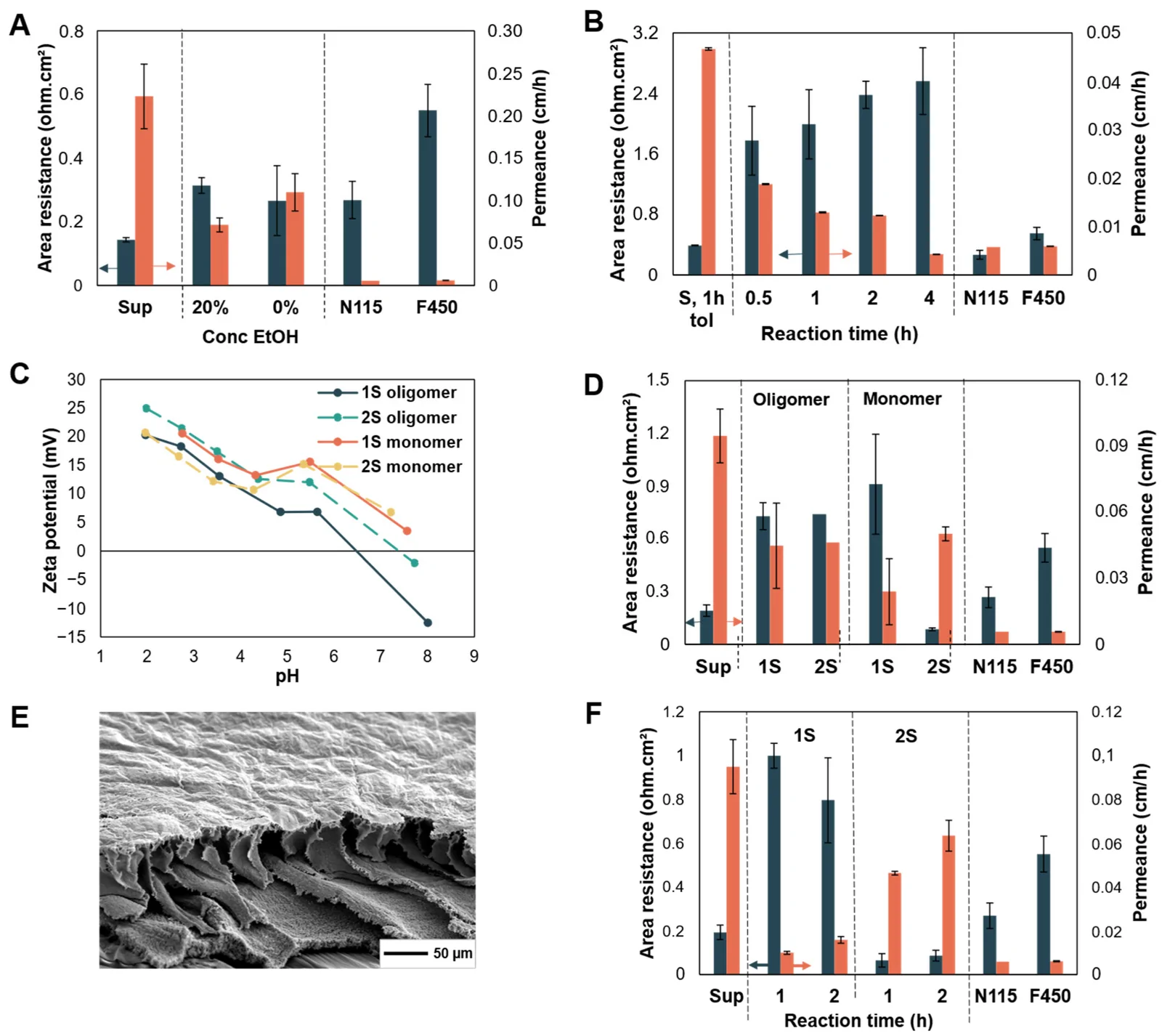
(**A**) Area resistance (blue, **left**) and vanadium permeance (red, **right**) of TFCs with and without ethanol as co-solvent in the aqueous phase, as compared to their support membrane (‘Sup’) and commercial references Nafion 115 and FAP 450. The dashed line is added for visual guidance. (**B**) Area resistance (blue, **left**) and vanadium permeance (red, **right**) of TFCs at varying interfacial reaction time, as compared to their support membrane (‘S’, toluene swelling accounted for) and commercial references Nafion 115 and FAP 450. Note that PVBC concentration is 10 wt% in all of the shown TFC membranes. The dashed line is added for visual guidance. (**C**) Zeta potential measurement of TFCs with varying crosslinker length (oligomer vs. monomer) and varying reaction steps (1S = 1 interfacial reaction, 2S = 2 steps of reactions). (**D**) Area resistance (blue, left) and vanadium permeance (red, right) of TFCs with varying crosslinker length (oligomer vs. monomer) and varying reaction steps (1S = 1 interfacial reaction, 2S = 2 steps of reactions), as compared to their support membrane (‘Sup’) and commercial references Nafion 115 and FAP 450. The dashed line is added for visual guidance. (**E**) SEM picture of cross section of TFC using a lab-made PVDF support. (**F**) Area resistance (blue, left) and vanadium permeance (red, right) of TFCs with TMHDA as crosslinker and of varying reaction steps (1S = 1 interfacial reaction, 2S = 2 steps of reactions) and varying reaction time, as compared to their support membrane (‘Sup’) and commercial references Nafion 115 and FAP 450. The dashed line is added for visual guidance. All area resistance measurements were performed with 0.5 M H_2_SO_4_.

The degree of miscibility of the aqueous phase and organic phase in interfacial synthesis determines the film properties. When both solvents are completely immiscible, a sharp interface is formed, restricting the reaction zone and thereby promoting the formation of a dense and thin top layer. In contrast, a less sharp interface may result in thicker top layers with a more crumpled morphology due to a more destabilized reaction phase, as seen in polyamide [53] and poly(epoxyether) [57] TFC membranes. The degree of miscibility can be tuned by varying the solvent of the organic phase. Another option is to use a co-solvent in the organic phase with a higher water miscibility or a co-solvent in the aqueous phase [53]. Here, ethanol was added as a co-solvent in the aqueous phase. This leads to a higher miscibility at the interface and an increased reactivity for top layer formation, as concluded from vial tests (Table S1). However, TFC membrane synthesis on PVDF supports shows a less clear difference (Figure 5A). While the TFC with less miscible reagent phases (0% EtOH) seems to have a slightly lower resistance and slightly higher vanadium permeability, the difference is not significant. To simplify the synthesis, the use of EtOH was omitted in further experiments.

#### 3.3.2. Concentration of Reagents

The concentration of the reagents can have a large effect on the resulting TFC. Hu et al. found that increasing the amine concentration facilitated the microphase separation [35], whilst Mandal et al. found that the conductivity drops at low amine concentration due to excessive water absorption inside the membrane at low crosslinking degrees [36]. To determine the relative effect of the PVBC-to-amine ratio in this system, two sets of reagent concentrations were tested. While using 10 wt% of DABCO-oligomer as the crosslinker in the aqueous phase, the PVBC concentration was doubled from 2.5 wt% to 5 wt%. However, this resulted in a decreased performance (Figure S4), with higher crossover at similar area resistance or higher area resistance at similar crossover. This indicates that an excess of PVBC does not improve top layer performance. The concentration of 2.5 wt% PVBC was thus used for further optimization. The concentration of amine was varied depending on the molecular weight, maintaining a constant molar amine-to-PVBC ratio: 10 wt% oligomer, 0.83 wt% DABCO and 1.41 wt% TMHDA.

#### 3.3.3. Reaction Time

An increased reaction time may lead to a denser and/or thicker top layer when crosslinking continues or when the reaction is not self-limiting [58]. In polyamide synthesis, reaction times vary around 1–2 min, where longer reaction times lead to rougher surfaces [53]. However, the reaction rate usually drops drastically after a certain time, and the film formation is considered self-limiting. This is because the formed top layer becomes so dense that the supply of monomers to the reaction zone is limited [20,59]. In current interfacial synthesis, where PVBC is crosslinked with oligomeric DABCO at the interfacial level (Figure 1A), no self-limiting behavior is seen in the tested time frame (Figure 5B). Instead, top layer formation continues to occur, shown by the increased area resistance and decreased vanadium crossover at higher reaction times. A thicker or denser top layer could explain this performance. At 4 h reaction time, the TFC membranes are able to reach a similar vanadium crossover to Nafion 115 but at a substantially higher area resistance. This is partially attributed to the support Nadir UV150 that was used during the TFC synthesis. Replacing Nadir UV150 with an alternative support would likely mitigate the high area resistance.

#### 3.3.4. Second Crosslinking Step

At lower reaction times, vanadium crossover is still too high while having a relatively high area resistance (Figure 5B). One possible solution could be to further crosslink the top layer to simultaneously densify this layer and induce more positive charges to repel the vanadium cations. Up to now, TFC membranes mainly relied on size exclusion to mitigate vanadium crossover [8,60]. A highly positively charged top layer could further suppress vanadium transport through combined size and Donnan exclusion [12]. Therefore, a second crosslinking step (‘2S’) was introduced, in which the aqueous amine solution is directly applied onto the top layer. This can act as a very targeted step and has proven beneficial in other TFC systems [61]. Here, the organic phase is discarded after the regular interfacial reaction (1st crosslinking step = 1S) and is replaced with aqueous amine solution (2nd crosslinking step = 2S) after washing to remove unreacted polymer. Thus, the amine can further densify the existing top layer and introduce additional positive charge without increasing the thickness. The higher volume of amine solution and the direct contact between amine and PVBC can allow for high incorporation of amine. However, for the DABCO-oligomer crosslinker, the performance of the 2S membrane is similar to 1S, even though more positive charges are indeed incorporated, as seen by the zeta-potential measurements (Figure 5C). An improved performance can be found when using other amine crosslinkers. In contrast to initial expectations, as the area resistance decreases, the vanadium crossover increases by implementing a second crosslinking step when using, e.g., monomeric DABCO as the crosslinker. This will be explained further in oncoming sections.

#### 3.3.5. Crosslinker Length

Originally, an oligomer of DABCO (Figure 1A) was used, consisting of nine DABCO molecules linked by eight hexane linkers (Figure S1). To further reduce the vanadium permeance, the DABCO-oligomer was replaced by the monomer (Figure 1B). It was expected that the top layer crosslinked by the monomer would have a denser network with less positive charge. On one hand, this denser network could reduce vanadium crossover. On the other hand, the decreased amount of positive charge in the top layer could lead to lower Donnan exclusion and thus higher vanadium crossover. As depicted in Figure 5D, the 1S membrane with a monomeric top layer has a slightly higher but similar area resistance to the 1S membrane with an oligomeric top layer, whilst also having a substantially lower vanadium crossover. SEM pictures of the TFC membrane with a monomeric top layer (Figure 5E) show the deposition of a very thin layer with a slightly crumpled texture. Zeta-potential measurements indicate a higher positive charge for TFC membranes made using the DABCO-monomer than for the DABCO-oligomer over the entire tested pH range (Figure 5C). This is in contrast with the hypothesis that the DABCO-oligomer, which contains more positive charges, would result in more charge incorporation. One possible explanation for this is that the oligomeric crosslinker, which is already charged in its uncrosslinked state, is more difficult to incorporate into the network due to the charge repulsion of nearby oligomers that already reacted. In addition, its reduced mobility could hamper reaching the necessary reactive sites. This would then result in a relatively loose top layer structure with low crosslinking degree, which might explain the relatively high vanadium crossover for these TFC membranes. On the other hand, the DABCO-monomer is indeed smaller and uncharged before reacting and can thus readily approach the PVBC top-layer network without being repelled by the already formed quaternary ammonium groups of the incorporated DABCO. Thus, a more crosslinked and denser network could be formed with monomeric DABCO, resulting in less vanadium crossover.

While the 2S crosslinking step shows no significant difference when using the DABCO-oligomer as a crosslinker, a strong decrease in resistance and increase in permeance is observed when implementing the DABCO-monomer as a crosslinker (Figure 5D). For the 2S monomer-based membrane, the area resistance extensively dropped to 0.086 Ω cm^2^ after 2 h of the second crosslinking step, while the vanadium crossover increased. This value is low compared to literature. Ji et al. prepared sulfonated polyamine TFCs, reaching 0.3 Ω cm^2^ in 3 M H_2_SO_4_. Sealing the pores of a PES/SPEEK support with a 180 nm thin polyamide layer resulted in a TFC resistance of 0.17 Ω cm^2^ in 0.5 M H_2_SO_4_ [8].

Zeta-potential measurements revealed that the 2S membrane showed a lower positive charge than the 1S membrane at low pH and a similar positive charge to the 1S membrane around neutral pH (Figure 5C). Remarkably, the area resistance of the 2S TFC membrane is lower than that of its support without the top layer. This could mean that the positively charged top layer enhances ion transport compared to the uncharged support or that the support degraded during synthesis of the top layer. However, given the limited net incorporation of additional positive charges, the former seems unlikely. A degradation of the support is noted, which will be further discussed in Section 3.3.7.

#### 3.3.6. DABCO vs. TMHDA

Thus far, all TFC membranes were made with DABCO or its oligomeric variant. Other diamines may be able to further improve the top layer. TMHDA, a linear diamine, shows good potential, with top layer formation in similar time frames as DABCO in vial tests. A particularly interesting aspect of TMHDA compared to DABCO is the equal reactivity of both amines. Due to the proximity of the two tertiary amine functions in DABCO, the second amine is less reactive once the first has been converted to a quaternary amine. This is reflected in the pK_b_’s of DABCO (resp. 5.2 and 11), which are used as a proxy for the nucleophilicity of the amine [62]. Due to the lower reactivity of the second amine, DABCO may be more likely to quaternize the PVBC polymer without crosslinking it (only reacting once instead of twice). TMHDA, on the other hand, shows only one pK_b_ at 3.3 [63], as the two amines are sufficiently distant to not be influenced by the other. Therefore, the likeliness of quaternization of the second amine is independent of the quaternization of the first. This makes TMHDA more likely to crosslink PVBC compared to DABCO. Additionally, the lower pK_b_ of TMHDA indicates a higher nucleophilicity. Theoretically, TMHDA could thus be a better crosslinker than DABCO. The results, however, show only a slight improvement of the TFC membranes made from TMHDA relative to DABCO (Figure 5D vs. Figure 5F). Specifically, the TFC prepared with TMHDA and two 1 h crosslinking steps exhibited a selectivity of 1.37 × 10^9^ mS.s cm^−3^, which is substantially higher than the 8.33 × 10^8^ mS.s cm^−3^ obtained for the TFC based on monomeric DABCO with two crosslinking steps of 2 h. Moreover, the TMHDA-based TFC surpassed the selectivity of FAP 450 (9.69 × 10^8^ mS.s cm^−3^), although Nafion remained the best-performing benchmark membrane, with a selectivity of 2.37 × 10^9^ mS.s cm^−3^. This insinuates that under current reaction conditions, the difference in reactivity between DABCO and TMHDA may be less pronounced than expected or that this is not the limiting factor for performance.

Variation in reaction time and synthesis steps (1S vs. 2S) shows that reaction durations of 1 h result in lower vanadium permeability compared to steps of 2 h, when TMHDA is used as a crosslinker (Figure 5F). This is in contrast to when the DABCO-oligomer was used. Implementing a second crosslinking step again leads to lower area resistance than the corresponding support and higher vanadium crossover, similar to the results when using the DABCO-monomer (Section 3.3.5). This will be further evaluated in Section 3.3.7.

#### 3.3.7. Degradation of Support During Second Crosslinking Step

**Figure 6:**
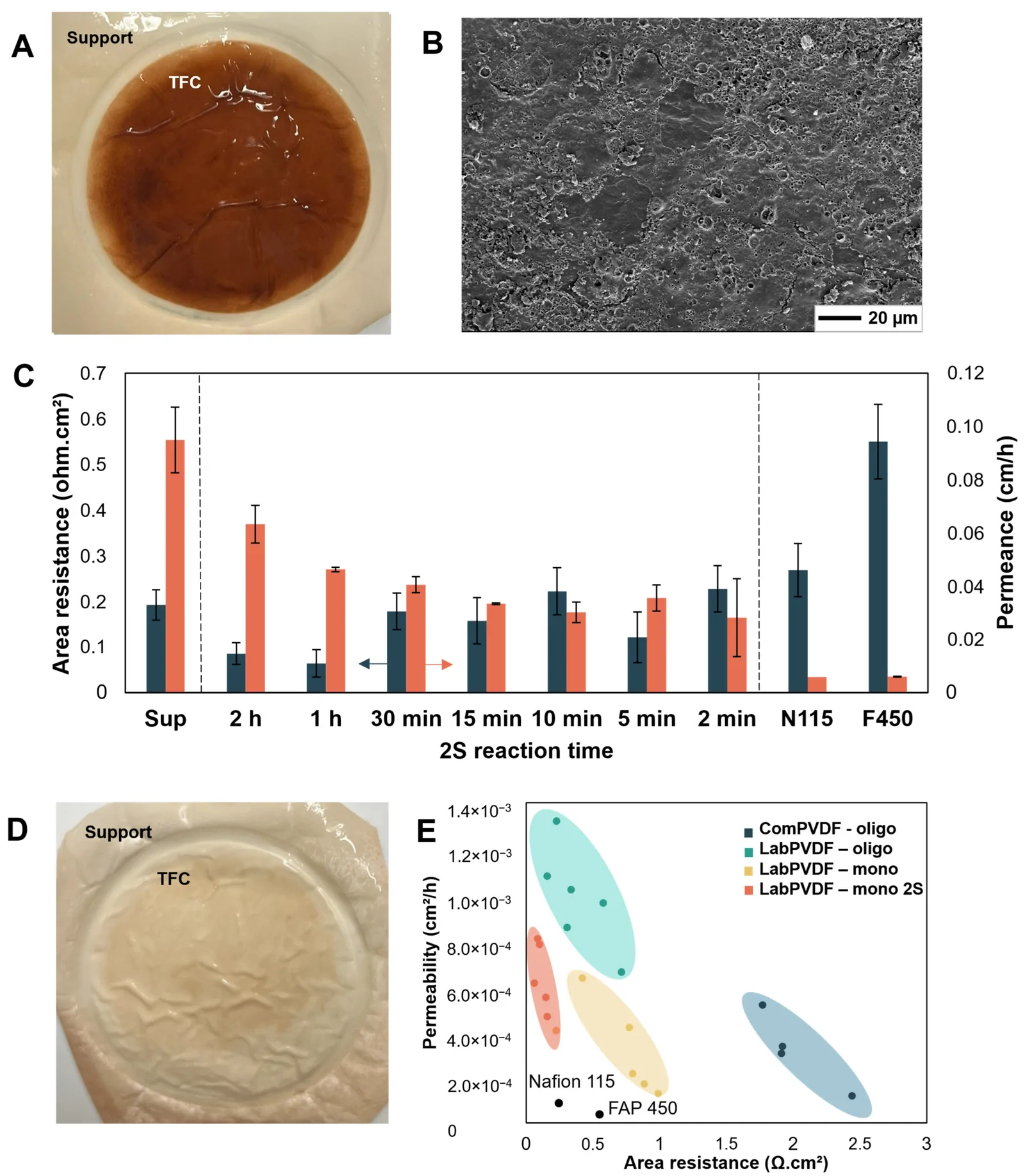
(**A**) Photo of TFC membrane prepared with monomer amine using 2S procedure, with 2 h reaction time. The TFC is formed within the circle. (**B**) SEM top-view image of 2S TFC membrane. (**C**) Area resistance (blue, left) and vanadium permeance (red, right) of TFC membranes from PVBC and TMHDA at decreasing reaction times of the 2S crosslinking step. The membranes are compared to their support (‘Sup’) and commercial membranes Nafion 115 and FAP 450. All area resistance measurements were performed in 0.5 M H_2_SO_4_. The dashed line is added for visual guidance. (**D**) Photo of TFC membrane prepared with monomer amine using 2S procedure, with 15 min duration time of second crosslinking step. (**E**) Area resistance vs. permeability of different types of TFCs described throughout this manuscript, grouped within their respective apparent trade-off regions. The dark blue group (bottom right) indicates TFCs prepared with DABCO-oligomer on commercial PVDF support Nadir UV150. The light-blue group (top left) indicates TFCs with DABCO-oligomer on lab-made PVDF support. The yellow group (bottom center) indicates TFCs with monomer amine on lab-made PVDF support prepared using 1 crosslinking step (1S). The red group (bottom left) indicates TFCs with monomer amine and a 2S crosslinking step on lab-made PVDF support.

In previous sections, 2S TFCs made from the DABCO-monomer and TMHDA show an increased vanadium crossover compared to their 1S TFCs, as well as an area resistance lower than their respective supports. This was not the case for oligomeric DABCO TFCs. Visually, the monomeric TFCs darken from off-white to brown (Figure 6A). During the 2S step, aqueous solutions of DABCO and TMHDA (0.83 wt% and 1.41 wt%, respectively) are added on top of the 1S membrane in an interfacial reaction set-up and heated to 50 °C to let the crosslinking reactions proceed. However, these aqueous amine solutions are alkaline and can thus cause dehydrofluorination of the PVDF support during this step, resulting in a more permeable support and thus contributing to decreased performance. 2S crosslinking with the DABCO-oligomer did not show such worsening, because the aqueous solution containing 10 wt% DABCO-oligomer was not as alkaline. While the monomeric amine solutions are more alkaline, the limited volume present in the PVDF support pores (during impregnation) was not sufficient to result in a visible PVDF dehydrofluorination during the first reaction step. SEM pictures of the TFC top layer show a disrupted morphology of the 2S membrane compared to the 1S membrane (2S in Figure 6B, 1S in Figure S5A). Although SEM pictures show a less affected morphology for 2S TMHDA (Figure S5B,C) than for 2S DABCO, TMHDA is not considered less disruptive than DABCO due to the slightly higher vanadium crossover, as well as the darker color of PVDF after second crosslinking with TMHDA.

It is hypothesized that the second crosslinking step may still be beneficial when limited in time and when balance between additional crosslinking of the top layer and dehydrofluorination of the support could be achieved. Shortened crosslinking times of 15 min to 45 min instead of 1 h do exhibit lower support degradation, as indicated by the lighter color of the support, which more closely resembles the color of the 1S PVDF support (2S at 15 min in Figure 6D; 2S at 1 h and 2S at 30 min in Figure S6). While the vanadium crossover of 2S membranes is still higher than that of 1S membranes, reducing the 2S crosslinking time enabled limitation of the vanadium crossover (Figure 6C). At the same time, the area resistance of 2S membranes remains lower than for the 1S membrane and increases with reduced 2S crosslinking reaction times. This is summarized in Figure 6E: while all other TFC membranes experience a trade-off of similar slope between vanadium crossover and area resistance, the 2S TFCs with varied reaction time experience a steeper trade-off slope, limiting the increase in resistance upon improving the vanadium crossover.

It can thus be hypothesized that further shortening the reaction time could lead to even better performance. However, this turned out to be difficult in practice for two reasons. On one hand, the membrane’s handling is done while still warm: the interfacial set-up containing the membrane is taken out of the oven, rinsed to remove unreacted PVBC, and supplied with fresh aqueous amine solution. After applying fresh amine solution, the still-warm interfacial set-up is assembled again and put back in the oven. This handling may take a few minutes, which becomes significant when reaction times become shorter. While this can already be a problem, the effect of the handling time is exacerbated because handling times may vary, as well as temperature due to handling. Longer handling times not only create a longer contact time with the amine at elevated temperatures, but the longer handling also intensifies the cooling down of the set-up. On the other hand, reaction times below 15 min may be insufficient to properly heat up the reaction mixture inside the interfacial set-up, and the time to reach the desired reaction temperature (i.e., 50 °C) will take up a significant chunk of the denoted reaction time. Both effects lead to an increased scattering of results when using second crosslinking times below 15 min.

**Figure 7:**
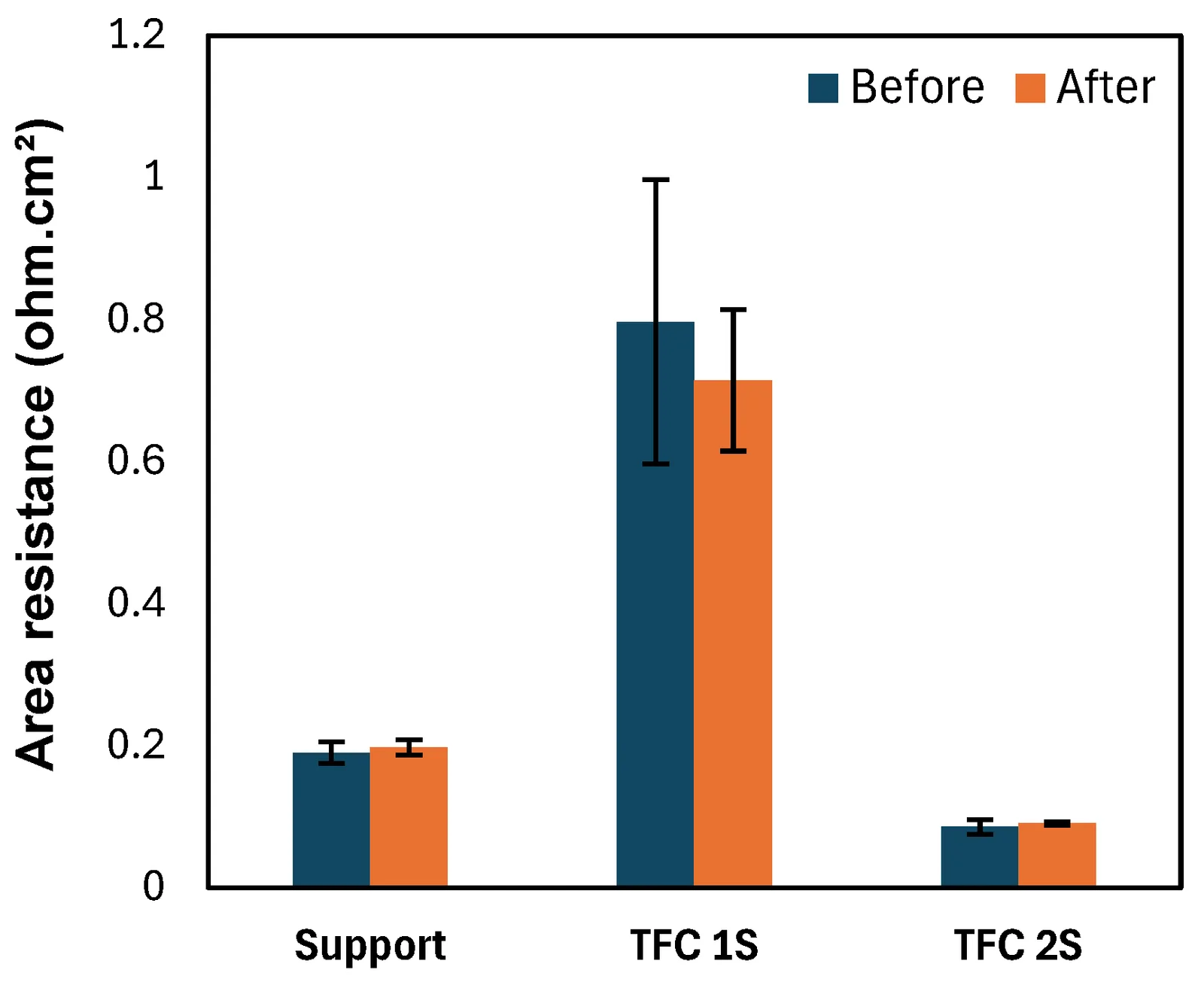
Area resistance of support, TFC 1S and TFC 2S using TMHDA as crosslinker before and after immersion in 0.5 M H_2_SO_4_ for 3.5 years.

Despite the limited stability of the support in alkaline conditions, the outstanding acidic stability of the TFC was demonstrated for 3.5 years in 0.5 M H_2_SO_4_ during an ex situ immersion test (Figure 7). The area resistances of the support, TFC 1S and TFC 2S after immersion were compared to the pristine membranes, showing no significant differences.

While the second crosslinking step holds the potential to improve the trade-off still experienced by TFC membranes, the true value of this top layer chemistry can only be unlocked in combination with an alkaline stable support. Future research should thus focus on finding a different support that is stable in a toluene, acid and alkaline environment. Preferably, this new support should be fluorine-free to align with the environmental concerns of fluorine-based chemistry. Promising alternatives that have been successfully employed in TFC membrane architectures previously include PES/SPEEK [8,60] blend supports and crosslinked poly(benzimidazole) [64]. Once a chemically robust support compatible with the crosslinked PVBC selective layer has been identified, single-cell VRFB testing will be essential to fully assess the potential of this novel selective layer chemistry. Finally, a techno-economic assessment of TFCs in VRFB systems is required to evaluate the practical viability of this emerging membrane concept [65].

## 4. Conclusions

Dense ion exchange membranes typically suffer from a performance trade-off, where reducing area resistance results in increased vanadium crossover. An alternative membrane morphology such as a TFC can break and/or shift this trade-off through the individual tailoring of the top layer and the support. In this work, TFC membranes prepared via an interfacial Menshutkin reaction between PVBC and an amine-based crosslinker were synthesized using a PVDF support.

Free-standing PVDF supports demonstrated drastically reduced area resistance compared to commercial PVDF due to the absence of a non-woven backing. A 17 wt% PVDF support was found optimal for the proposed interfacial reaction. Higher PVBC-to-amine ratios were found to increase the area resistance with limited benefit to the vanadium crossover. The use of monomeric bifunctional amine crosslinkers, such as DABCO and TMHDA, was preferred over oligomeric amine crosslinkers, as this led to lower vanadium crossover, caused by improved top layer density and enhanced charge incorporation. A second crosslinking step was proven promising to break the trade-off between resistance and selectivity, but it ultimately resulted in breakdown of the PVDF support due to the high pH of the amine solution. Future research should thus focus on finding a more suitable support to unlock the true potential of this novel TFC membrane.

## Data Availability

The raw data supporting the conclusions of this article will be made available by the authors on request.

## Supplementary Materials

The following supporting information can be downloaded at: https://www.mdpi.com/article/10.3390/membranes16090291/s1, Figure S1: ^1^H-NMR spectrum of DABCO-oligomer; Figure S2: SEM image of (A) Nadir UV150 and (B) TFC with PVBC/DABCO-oligomer top layer on Nadir UV150; Figure S3: Image of free-standing PVDF membranes at increasing HDA crosslinking times. From left to right, pristine, 15 min crosslinking, 30 min crosslinking, 45 min crosslinking, 1 h crosslinking; Figure S4: Area resistance and permeance of TFC membranes prepared using varying reagent concentrations (2.5 wt% PVBC: 10 wt% DABCO-oligomer vs. 5 wt% PVBC: 10 wt% DABCO-oligomer) and at varying reaction times (2h vs. 4h). The composites are compared against their support (‘Sup’); Figure S5: (A) SEM top-view image of 1S TFC membrane made with DABCO monomer. (B) SEM top-view image of 2S TFC membrane made with TMHDA. (C) SEM top-view image of 1S TFC membrane made with TMHDA; Figure S6: (A) Photo of 2S TFC membrane with TMHDA, with 1 h duration of 2S crosslinking step. (B) Photo of 2S TFC membrane with TMHDA, with 30 min duration of 2S crosslinking step. In both cases, the TFC is formed within the circle; Table S1: Descriptive results of vial tests of PVBC and DABCO-oligomer. 10wt% DABCO-oligomer in water and 10wt% PVBC in toluene were put in a glass vial (calmly, as to not disturb the formed interphase) and left to react. The formation of a reacted layer at the interphase was checked intermittently.
