# Supplementary material for: Shifting the Redox-Flow Battery Trade-Off with Amine-Crosslinked PVBC Thin-Film Composite Membranes
Source: Membranes (Basel). 2026 Aug 31;16(9):291. doi: 10.3390/membranes16090291 (PMC13609740; doi:10.3390/membranes16090291)
Supplement: Supplementary file 1 [file membranes-16-00291-s001.zip › membranes-4487634-supplementary.pdf]

# Supplementary information: Shifting the Redox-Flow Battery Trade-off with Amine-crosslinked PVBC Thin Film Composite Membranes

Chiari Van Cauter [1], Maarten Cools [1], Yun Li and Ivo Vankelecom *

Membrane Technology Group (MTG), Division cMACS, Faculty of Bioscience Engineering, KU Leuven, Celestijnenlaan 200F, Box 2454, 3001 Leuven, Belgium

*Correspondence: ivo.vankelecom@kuleuven.be

[1]Co-first authors

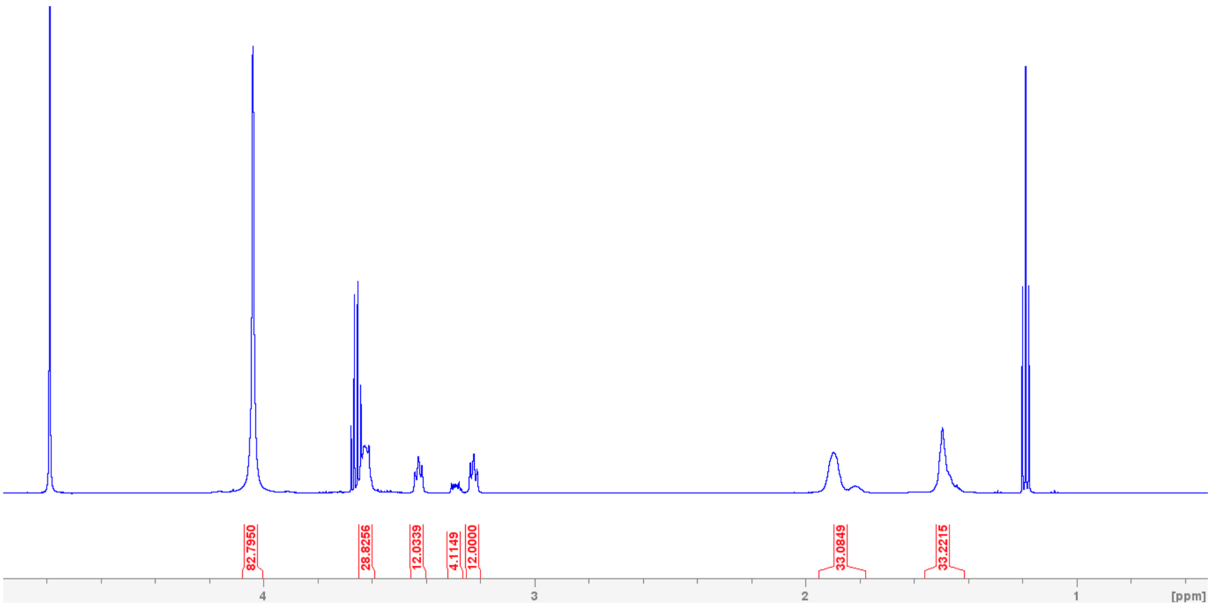


*Figure S1: $^{1}$H-NMR spectrum of DABCO-oligomer.*

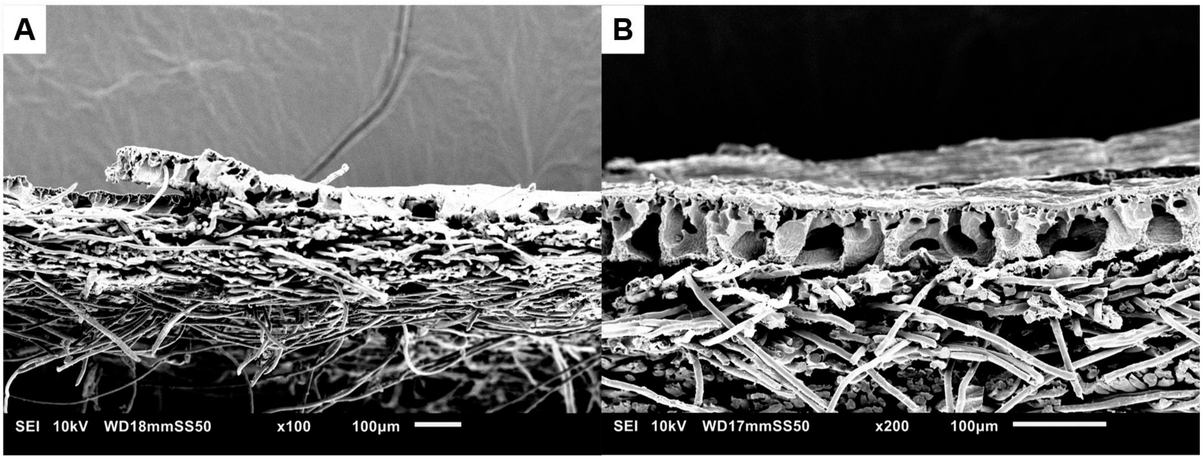


*Figure S2: SEM image of (A) Nadir UV150 and (B) TFC with PVBC/DABCO-oligomer top layer on Nadir UV150.*

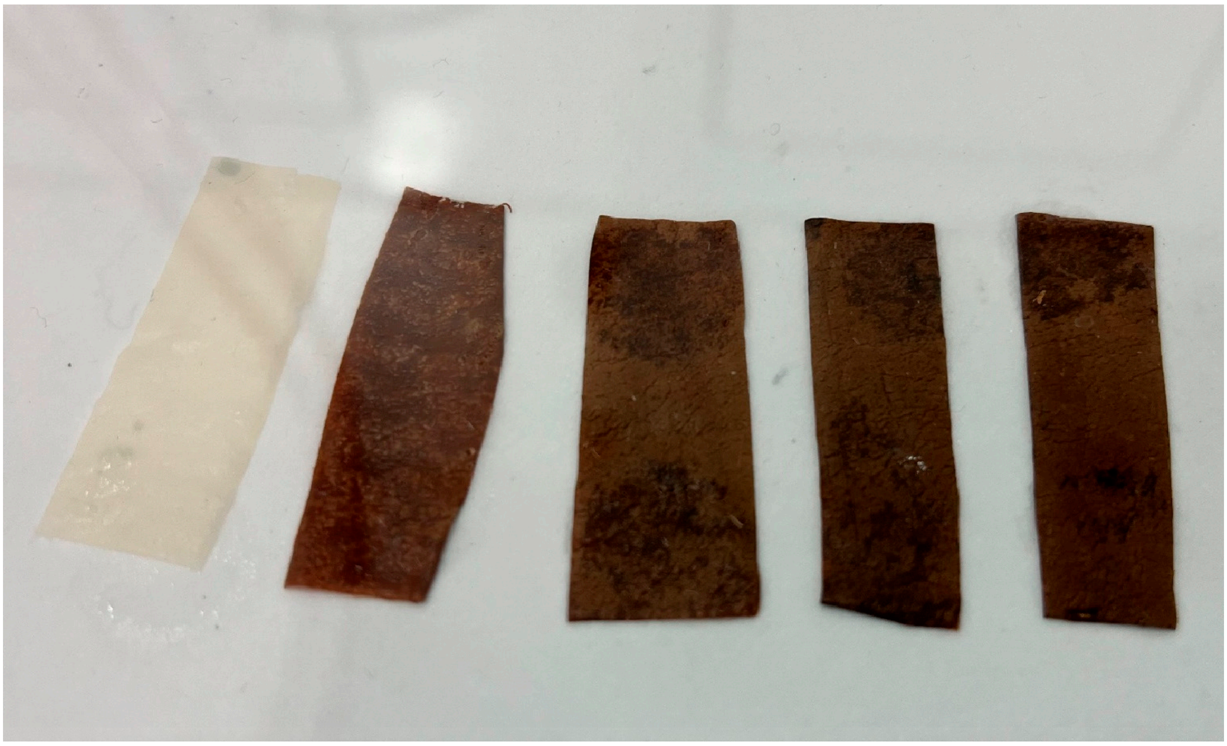

*Figure S3: Image of free-standing PVDF membranes at increasing HDA crosslinking times. From left to right, pristine, 15 min crosslinking, 30 min crosslinking, 45 min crosslinking, 1 h crosslinking.*

*Table S1: Descriptive results of vial tests of PVBC and DABCO-oligomer. 10wt% DABCO-oligomer in water and 10wt% PVBC in toluene were put in a glass vial (calmly, as to not disturb the formed interphase) and left to react. The formation of a reacted layer at the interphase was checked intermittently.*

| | **EtOH in water phase** | **No EtOH in water phase** |
|---|---|---|
| **Room temperature (ca. 22°C)** | With concentration 10% EtOH, 15% EtOH, 20% EtOH:<br>No layer formed (after 6 days) | No layer formed (after 8 days) |
| **50°C** | With concentrations 10% EtOH, 15% EtOH, 20% EtOH:<br>Thinly-formed layer at the interphase (after 2 days)<br>Thinly-formed layer at the interphase (after 6 days)<br>Thickly-formed layer at the interphase (after 9 days)<br>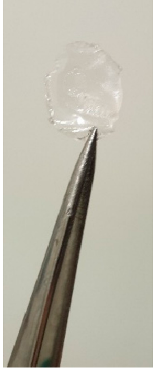: removed from vial<br>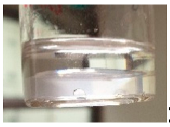: shorter time-frame<br>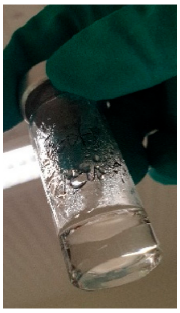: extended time-frame | No layer formed (after 16 days) |

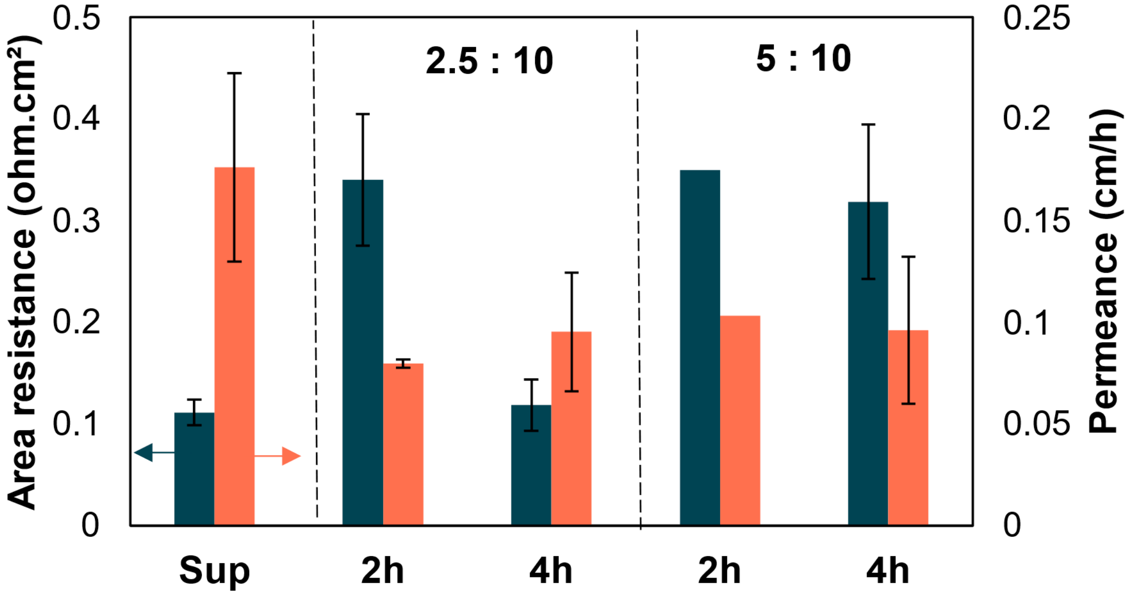


*Figure S4: Area resistance and permeance of TFC membranes prepared using varying reagent concentrations (2.5 wt% PVBC : 10 wt% DABCO-oligomer vs 5 wt% PVBC : 10 wt% DABCO-oligomer) and at varying reaction times (2h vs 4h). The composites are compared against their support ('Sup').*

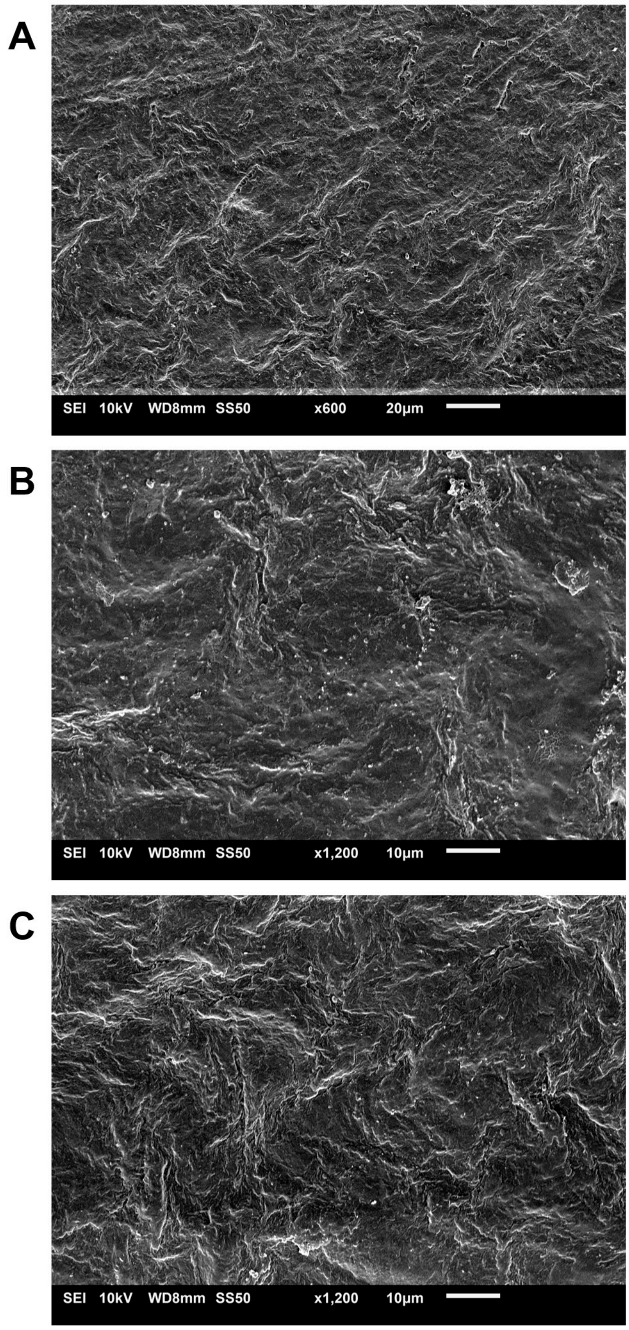


*Figure S5: (A) SEM top-view image of 1S TFC membrane made with DABCO monomer. (B) SEM top-view image of 2S TFC membrane made with TMHDA. (C) SEM top-view image of 1S TFC membrane made with TMHDA.*

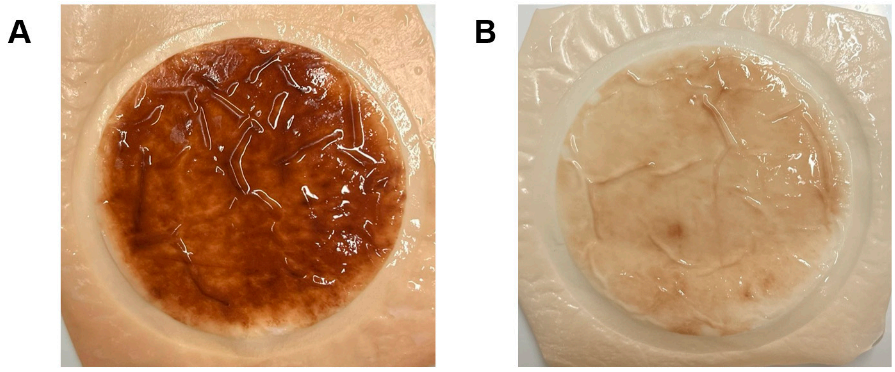


*Figure S6: (A) Photo of 2S TFC membrane with TMHDA, with 1 h duration of 2S crosslinking step. (B) Photo of 2S TFC membrane with TMHDA, with 30 min duration of 2S crosslinking step. In both cases, the TFC is formed within the circle.*
